# Enhancing Electrochemical Properties of Vitreous Materials Based on CaO–Fe_2_O_3_–Fe–Pb and Recycled from Anodic Plate of a Spent Car Battery

**DOI:** 10.3390/ma18092017

**Published:** 2025-04-29

**Authors:** Delia Niculina Piscoiu, Simona Rada, Horatiu Vermesan

**Affiliations:** 1 Faculty of Materials and Environmental Engineering, Technical University of Cluj-Napoca, 400641 Cluj-Napoca, Romania; deliapiscoiu95@gmail.com (D.N.P.); horatiu.vermesan@imadd.utcluj.ro (H.V.); 2 National Institute for Research and Development of Isotopic and Molecular Technologies, 400293 Cluj-Napoca, Romania

**Keywords:** anodic plate, recycling, vitroceramics, structure, electrochemical performance

## Abstract

This paper presents a novel approach for the recycling of spent anodic plates from lead-acid batteries through the melt quenching method using iron and calcium oxides and iron powder. The resulting recycled samples, with a 3CaO·5Fe_2_O_3_·xFe·(92 − x)Pb composition, where x = 0, 1, 3, 5, 8, 10, 15, and 25% mol Fe, were characterized and analyzed in terms of their electrochemical performance. X-ray diffractograms show vitroceramic structures with varied crystalline phases. Analysis of the IR (infrared spectra) data shows a decrease of sulphate units due to doping with iron content. The ultraviolet–visible (UV-Vis) and electron spin resonance (ESR) data reveal the presence of Fe^3+^ ions with varied coordination geometries. Cyclic and linear sweep voltammograms demonstrate that the samples with 8 and 10% Fe exhibit superior electrochemical performance compared to other vitroceramics. The electrochemical impedance spectroscopy measurements indicate that the sample with 8% Fe had lower resistance compared to other analogues and had enhanced electrical conductivity.

## 1. Introduction

Recent studies have highlighted the necessity of efficient and environmentally friendly processes for recycling lead-acid batteries. Improper disposal of these batteries can result in the release of lead and other pollutants into the environment, contaminating the soil, water, and air [1].

The importance of recycling lead-acid batteries cannot be underestimated. Recycling not only prevents the release of harmful substances into the environment but also provides a sustainable source of raw materials for the production of new batteries, reducing dependence on primary mineral resources. However, current recycling technologies often face challenges such as low process efficiency and the risk of secondary pollution, which hinder their large-scale application.

The compounds CaO, Fe, and Fe_2_O_3_ play significant roles in the recovery and treatment of materials from spent lead–acid batteries.

The presence of calcium oxide is particularly important in the recycling of used lead-acid batteries, as it contributes to the neutralization of sulfuric acid through the chemical reaction that forms calcium sulfate (CaSO_4_) [2]. The addition of calcium enhances the mechanical properties of lead alloys; however, it does not play a catalytic role and may reduce the corrosion resistance of lead anodes [3].

Iron-based compounds can be used as flux materials in the smelting process of lead-rich fractions, facilitating the separation and recovery of lead metal [4]. Metallic iron is also used in the recycling process of used automotive batteries, primarily as a reducing agent in lead smelting. It reacts with lead oxides to produce metallic lead and iron oxide. Iron is a relatively inexpensive and abundant metal, making it a cost-effective option for this process.

Additionally, iron oxide compounds can also be used as absorbents or stabilizing agents in the treatment of hazardous waste streams generated during the recycling process. This helps minimize the environmental impact of recycling operations and ensures the safe disposal of any remaining residual materials [5,6].

The improper disposal and the relatively short lifespan (approximately 4–6 years) of the batteries is leading to an increasing of amount of waste in more and more landfills. The statistical data indicate that China will generate approximately 45,000 tons of spent batteries by 2025 [7]. Consequently, the recycling of spent batteries becomes imperative. Batteries are widely used in the automotive sector and various industrial applications. At the end of the life cycle, batteries become significant sources of toxic waste. Their efficient recycling contributes to the recovery of valuable materials and the conservation of natural resources. On the other hand, the recycling process reduces the volume of waste and the negative ecological impact associated with the inappropriate disposal of hazardous products, protecting public health and the environment.

In this paper, new recycled and iron-doped materials with a 3CaO·5Fe_2_O_3_·xFe·(95-x)Pb composition, where x = 0, 1, 3, 5, 8, 10, 15, and 25 mol% Fe, will be prepared and investigated for their application as active electrodes in lead-acid batteries. The study aims to analyze how these additives influence the electrochemical performance of electrodes used in lead-based batteries. This approach represents a significant contribution in the field of sustainability and in the development of new solutions for obtaining secondary lead compounds.

Iron (III) oxide, Fe_2_O_3_, plays a crucial role in the improvement of battery performance by promoting more efficient charge–discharge cycles and reducing the formation of sulfate layers on lead plates. The sulphatization phenomenon degrades the performance of lead-acid batteries. Doping with calcium oxide, CaO, contributes to enhancing the hardness and structural stability of the material, to prevent rapid corrosion and to extend of electrode’s lifespan under intensive use conditions [8,9].

Studies have shown that doping with metallic oxides, such as Fe_2_O_3_, enhances the thermal and electrochemical stability of these electrodes, thereby optimizing the stored energy density and life cycle duration [9,10].

## 2. Experiment

Samples with 3CaO·5Fe_2_O_3_·xFe·(95-x)Pb composition, where x = 0, 1, 3, 5, 8, 10, 15, and 25 mol% Fe, were prepared by the melt quenching method using a spent anodic plate (as the source of Pb) from a discarded car battery, iron, iron oxide, and calcium oxide powders (Sigma Aldrich, Vienna, Austria) as the raw materials.

The chemical formulas in molar percentages of the prepared samples are 3CaO·5Fe_2_O_3_·92Pb, 3CaO·5Fe_2_O_3_·1Fe·91Pb, 3CaO·5Fe_2_O_3_·3Fe·89Pb, 3CaO·5Fe_2_O_3_·5Fe·87Pb, 3CaO·5Fe_2_O_3_·8Fe·84Pb, 3CaO·5Fe_2_O_3_·10Fe·82Pb, 3CaO·5Fe_2_O_3_·15Fe·77Pb, and 3CaO·5Fe_2_O_3_·25Fe·67Pb.

All substances were weighed in stoichiometric amounts according to the chemical formula mentioned above using an analytical balance. The mixture was finely ground and homogenized in an agate mortar to ensure the uniform distribution of the components.

After homogenization, the mixture was placed in a ceramic crucible and heated in an electric furnace. The host matrix was melted at 1050 °C and 1150 °C. After a synthesis time of 10 min, the crucibles were removed from the furnace, and the molten material was rapidly poured onto a stainless-steel plate. This thermal shock allows the rapid vitrification of the material.

The optimal temperature for the host matrix was selected to be 1150 °C due to the obtaining of better homogeneity in the final product. The mentioned system was prepared at 1150 °C and kept in the electric furnace for 10 min, a sufficient duration for all components to reach a fully molten and homogeneous state.

The structure of the obtained materials was analyzed using the following characterization methods: X-ray diffraction (XRD), Fourier transform infrared spectroscopy (FTIR), ultraviolet–visible spectroscopy (UV-Vis), and electron spin resonance (ESR).

X-ray diffraction measurements were performed using a Shimadzu XRD-6000 diffractometer equipped with a graphite monochromator for a copper anode tube (λ = 1.54 Å). X-ray diffraction analysis enables the determination of the amorphous or crystalline nature of the samples, while the identification of crystalline phases from the diffractograms was carried out using Match! Software, version 1.0 (Crystal Impact, Bonn, Germany).

Infrared absorption spectra were recorded at room temperature using a JASCO 6200 Fourier transform infrared (FTIR) spectrometer.

UV-Vis absorption spectra were obtained with a Perkin-Elmer Lambda 45 spectrophotometer, equipped with an integrating sphere, ensuring a band position accuracy of ±2 nm. For IR and UV-Vis spectral recordings, pellets were prepared by mixing potassium bromide (KBr) and the sample in a 150:1 mass ratio.

Electron spin resonance (ESR) spectroscopy measurements were conducted at room temperature, in the X-band frequency range (9.52 GHz), using a Bruker ELEXSYS 500 spectrometer.

For cyclic voltammetry, linear sweep voltammetry, and electrochemical impedance spectroscopy experiments, an electrochemical cell with three electrodes was used, immersed in a 5 M sulfuric acid electrolyte solution. A calomel electrode (Hg/Hg_2_Cl_2_/KCl) was used as the reference electrode, while platinum was used as the counter electrode. The working electrode consisted of the prepared material. Electrochemical measurements were carried out using a Metrohm Autolab PGSTAT 302N potentiostat/galvanostat, equipped with Nova 1.11 software (EcoChemie, Ultrecht, The Netherlands).

## 3. Results and Discussions

**Figure 1:**
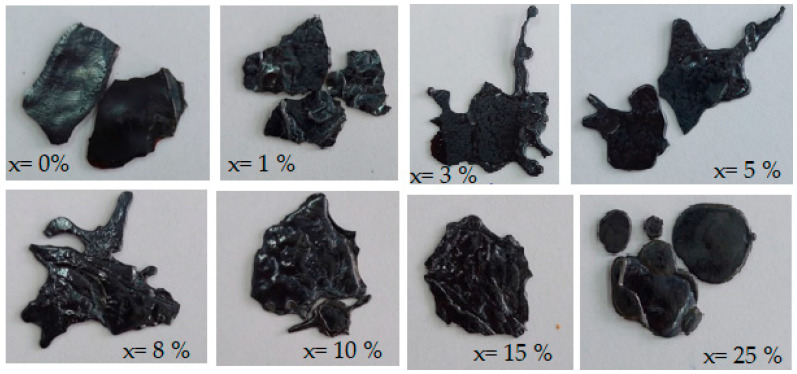
FujiFilm photos of prepared samples in the vitreous system with the 3CaO·5Fe_2_O_3_·xFe·(92 − x)Pb composition, where x = 0, 1, 3, 5, 8, 10, 15 and 25 mol% Fe, prepared at 1150 °C.

Figure 1 shows the prepared samples with the 3CaO·5Fe_2_O_3_·xFe·(92 − x)Pb composition, where x = 0, 1, 3, 5, 8, 10, 15, and 25% mol Fe. All samples exhibit a black-gray color. The glass-ceramic appearance is an important characteristic that suggests a stable structure and could contribute to superior performance when it is used as an electrode in lead-acid batteries. Doping with CaO and Fe_2_O_3_ is expected to modify the crystallographic structure of the lead anodic plate, thereby influencing its electrochemical properties, such as energy storage capacity and cyclability [8].

### 3.1. Analysis of X-Ray Diffraction (XRD)

**Figure 2:**
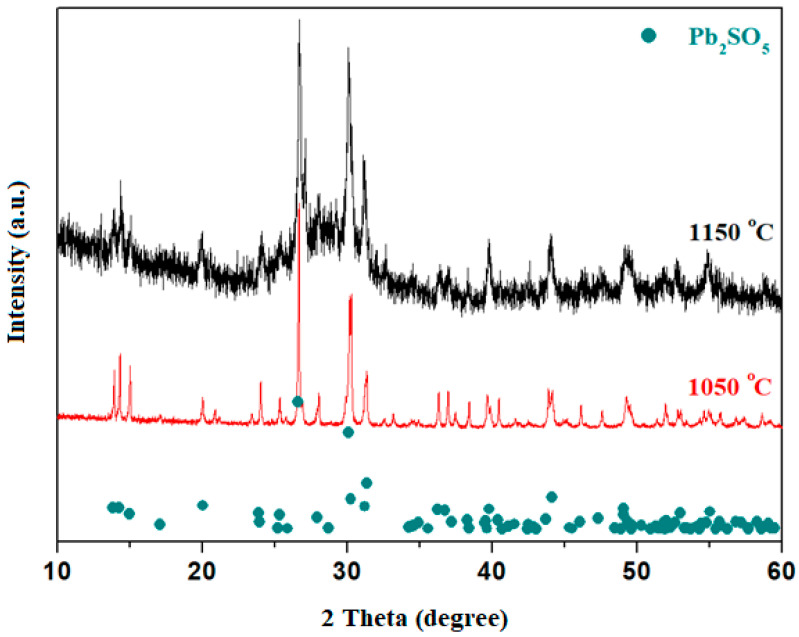
XRD diffractograms of 3CaO·5Fe_2_O_3_·92Pb sample prepared at 1050 °C and 1150 °C.

The XRD patterns of the host matrices with the 3CaO·5Fe_2_O_3_·92Pb chemical formula prepared at 1050 °C and 1150 °C are presented in Figure 2. Analysis of the diffractograms indicates that both samples are vitroceramics consisting of Pb_2_SO_5_ ≡ PbO·PbSO_4_ crystalline phase.

The diffraction peaks located at 26.7° and 30.09°, with 100% and 75% intensity, respectively, corresponding to the Pb_2_SO_5_ crystalline phase, are significantly more intense for the sample prepared at 1050 °C compared to the one prepared at 1150 °C. Therefore, the Pb_2_SO_5_ crystalline phase is the major phase in the glass-ceramic material prepared at 1050 °C, while the amount of this phase decreases in the sample prepared at 1150 °C.

The amount of Pb_2_SO_5_ crystalline phase has an increased content in the sample prepared at 1050 °C. The host matrix prepared at 1150 °C exhibits two broad halos characteristic of an amorphous structure, over which are superimposed diffraction peaks corresponding to the predominant Pb_2_SO_5_ oxo-sulfate crystalline phase.

Analysis of the XRD data suggests that the sample prepared at 1150 °C is more suitable for doping with metal ions because it contains a lower amount of the oxo-sulfatized crystalline phase.

**Figure 3:**
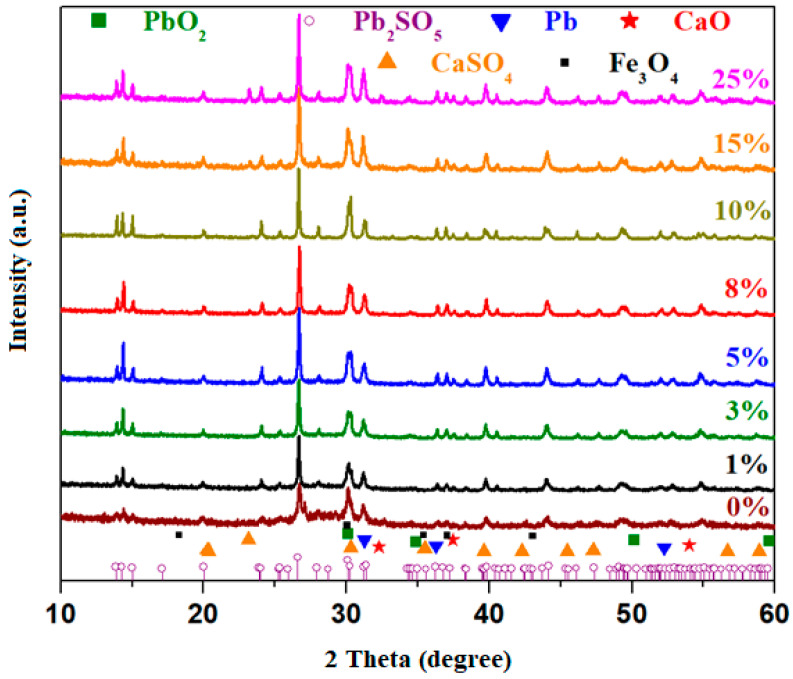
XRD diffractograms of 3CaO·5Fe_2_O_3_·xFe·(92 − x)Pb samples, where x = 0, 1, 3, 5, 8, 10, 15 and 25 mol% Fe.

The X-ray diffraction patterns of the materials prepared with the 3CaO·5Fe_2_O_3_·xFe·(92 − x)Pb composition, where x = 0, 1, 3, 5, 8, 10, 15, and 25 mole% Fe, are illustrated in Figure 3. The diffractograms reveal diffraction peaks located at 14.37°, 20.03°, 24.03°, 25.35°, 26.67°, 28.04°, 30.1°, 31.20°, 36.98°, 39.69°, 44.03°, 49.28°, and 54.80°.

The host matrix with the 3CaO·5Fe_2_O_3_·92Pb composition prepared at 1150 °C exhibits a glass-ceramic structure consisting of the Pb_2_SO_5_ crystalline phase as the major component. In smaller quantities, additional crystalline phases include: Fe_3_O_4_ (magnetite) crystalline phase with a cubic structure, with the diffraction peak of the highest intensity situated at 2θ = 30.07°; PbO_2_ (lead dioxide) with a cubic structure, with the main diffraction peak at 30.08°; metallic lead with a cubic structure, with the primary diffraction peak at 31.2°; and traces of CaO (calcium oxide) crystalline phase with cubic structure and diffraction peak at 2θ = 32.2°.

Doping with a higher iron content of up to 25 mol% results in an increase in the intensity of the diffraction peaks corresponding to the Pb, PbO_2_, and Fe_3_O_4_ crystalline phases. For samples with x ≥ 8 mol% Fe, a smaller diffraction peak appears at 2θ = 23.2°, attributed to the CaSO_4_ crystalline phase. This peak exhibits slightly higher intensity in the samples containing 15 and 25 mol% Fe. The XRD data suggest that at higher dopant levels, calcium ions interact with free sulfate groups released from lead ions. It is observed that glass-ceramics with a higher iron content are enriched in the PbO_2_, Fe_3_O_4_, and Pb crystalline phases. This structural evolution can be explained by lead and iron atoms exhibiting a stronger affinity for oxygen atoms, which lead to the formation of lead dioxide (PbO_2_) and magnetite (Fe_3_O_4_).

### 3.2. Infrared Spectra

**Figure 4:**
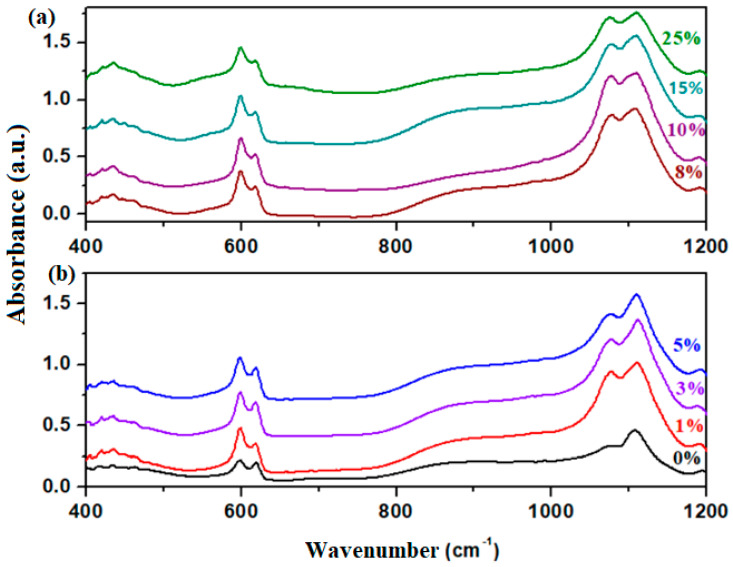
FTIR spectra of 3CaO·5Fe_2_O_3_·xFe·(95-x)Pb samples: (**a**) x = 8, 10, 15, and 25 mol%, and (**b**) x = 0, 1, 3, and 5 mol% Fe.

FTIR spectroscopy was used to examine the effect of various iron levels on the vibrational modes and structural modifications of the vitreous system. The FTIR spectra of the prepared glass-ceramics are illustrated in Figure 4.

The FTIR spectra show changes in the intensity of the IR bands with the increasing of dopant levels. In the domain between 400 and 1200 cm^−1^ can be observed three regions of significant IR bands corresponding to the vibrational modes of various structural units of lead and iron [11]. The first IR band region located between 400 and 500 cm^−1^ is associated with an overlap of IR bands originating from the stretching vibrations of Fe–O bonds in [FeO_6_] structural units [12,13] and the bending vibrations of Pb–O–Pb angles in [PbO_4_] tetrahedral units [14]. With the addition of iron content into the host matrix, an increase in the intensity of the IR band centered at 430 cm^−1^ is observed, suggesting an enrichment of the glass-ceramic material with crystalline phases of lead and iron, specifically PbO_2_ and Fe_3_O_4_, in agreement with the XRD data.

The second region of IR bands with medium intensity is located between 500 and 750 cm^−1^ and includes two maxima centered at 600 and 620 cm^−1^. The first IR band is attributed to stretching vibrations of the S–O bonds in sulfate units, while the second IR band corresponds to sulfite units [15]. With the addition of iron levels up to 10%, an increase in the intensity of the IR bands centered at 600 and 620 cm^−1^ is observed. However, for higher dopant contents, the intensity of these IR bands decreases. This structural evolution indicates a reduction in the number of sulfate and sulfite units in the glass-ceramic matrix at dopant levels above 10%. A partial conversion mechanism of the oxo-sulfatized Pb_2_SO_5_ phase into the CaSO_4_ and PbO_2_ crystalline phases can be proposed based on the XRD data.

The last region of IR bands of higher intensity is situated between 750 and 1200 cm^−1^ and is characterized by three maxima centered at 870, 1075, and 1100 cm^−1^. The IR band centered at 870 cm^−1^ is attributed to an overlap of the Pb–O stretching vibrations in the [PbO_3_], [PbO_4_], and [PbO_6_] structural units [14,15,16]. The intensity of this IR band increases gradually with doping up to x ≤ 5 mol% Fe, further decreases for the 8% Fe, and reaches a minimum value at x = 10% Fe. Afterward, the intensity of the IR band reaches a maximum value at higher doping levels up to 15%, followed by another decrease for the 25% Fe sample. For the 10% Fe sample, the number of [PbO_n_] structural units is reduced, indicating a lower degree of polymerization of the host matrix. This hypothesis supports the idea that lead is associated with oxygen and sulfate ions in the Pb_2_SO_5_ crystalline phase. At higher concentrations, calcium ions interact with sulfate ions, causing the liberation of lead atoms, which become network formers by their combination with oxygen atoms. For the sample with 25% Fe, the decrease of intensity of the IR bands is associated with a lower degree of polymerization of the host matrix compared to the sample with x = 15% Fe. The lower polymerization degree suggests an increased structural disorder due to the formation of larger CaSO_4_ amounts. The increase of Fe content in the host matrix leads to a breakdown of more structures originating from the Pb_2_SO_5_ crystalline phase.

The last IR bands situated between 1075 and 1100 cm^−1^ correspond to the stretching vibrations of S–O bonds in sulfate units [13]. With the addition of dopant levels up to 10% Fe, the intensity of these IR bands reaches a maximum and then decreases for the samples with 15% and 25% Fe. The number of sulfate units decreases with the addition of higher iron contents. The mechanism responsible for these structural evolutions can be described as follows: (i) for samples with 0 ≤ x ≤ 10 mol% Fe, the content of oxo-sulfatizated phases reaches a maximum value, and the iron atoms act as a network former; (ii) for samples with x > 10 mol% Fe, iron becomes a network modifier because it leads to the breakdown of lead oxo-sulate phases, the formation of calcium sulfate phases, and an increase in the polymerization degree of lead atoms.

By doping with Fe contents, new crystalline phases appear, which imply the rearrangements of the structural units with calcium, iron, or lead ions. The calcium and iron atoms have smaller electronegativity (of 1.83 and 1.00, respectively) than the lead atoms (2.33). The covalence of Pb–O bond is stronger than that of Fe–O and Ca–O bonds. The lead and iron atoms have a strong affinity towards oxygen atoms. The increase of intensity of the IR band centered at about 470 cm^−1^ show the enhancement of bending vibrations of Pb–O–Pb angles in [PbO_4_] tetrahedral units due to the conversion of [PbO_3_] into [PbO_4_] structural units by doping. The calcium ions can substitute the lead ions in some sulphate units. The excess of oxygen atoms can be accommodated in the host matrix by the formation of Fe_3_O_4_ crystalline phase. For the sample with 25 mol% Fe, the decrease of intensity of the IR bands situated in the region between 400 and 1200 cm^−1^ show that the connectivity and polymerization grade of the host matrix attain their maximum value. For their analogues at smaller contents, the IR bands are sharper and intense, indicating a higher grade of depolymerization of the network.

### 3.3. Ultraviolet–Visible (UV-Vis) Spectra

**Figure 5:**
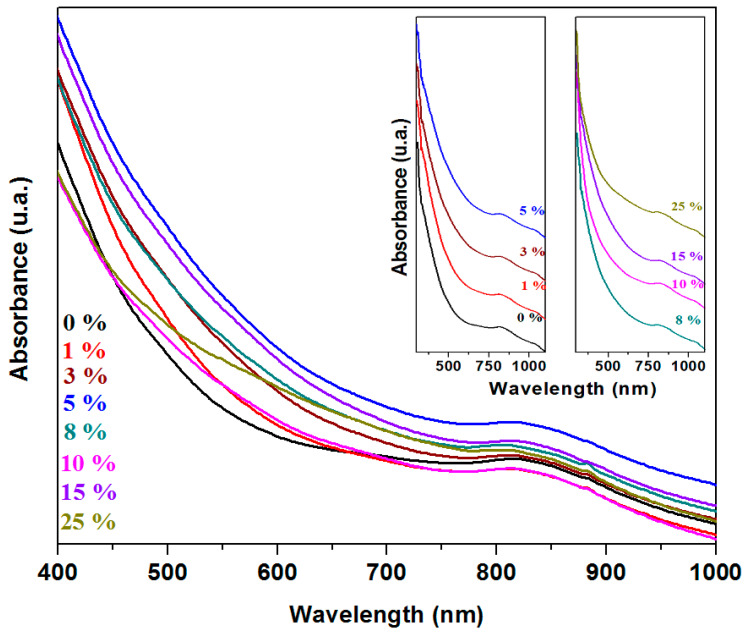
UV-Vis spectra of studied samples.

The UV-Vis spectra of the recycled and doped system with the 3CaO·5Fe_2_O_3_·xFe·(92 − x)Pb composition are shown in Figure 5. The Pb^2+^ ions exhibit characteristic UV-Vis bands at 320 nm. The Fe^3+^ ions have absorption bands in the region between the 400–450 nm and 700 nm regions, while the Fe^2+^ ions produce an absorption band in the 780–900 nm range [16,17].

With the increase of doping levels, the intensity of the UV-Vis bands increases across the entire 300–1100 nm range, reaching maximum values for the sample with 5% and 15% Fe and dropping sharply for the 10% Fe. These structural evolutions indicate that the concentration of lead and iron ions reaches a maximum value for the samples with 5% and 15% Fe and a minimum value for 10% Fe. The sample with a higher content of oxosulfatized phase (x = 10%) exhibits a lower number of electronic transitions due to Pb^2+^ and Fe^2+^/Fe^3+^ ions.

### 3.4. Electron Spin Resonance (ESR) Spectra

**Figure 6:**
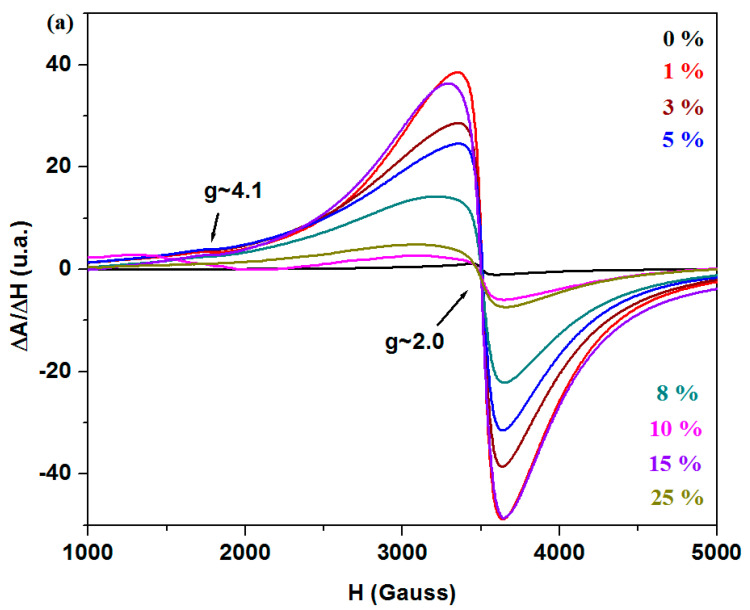

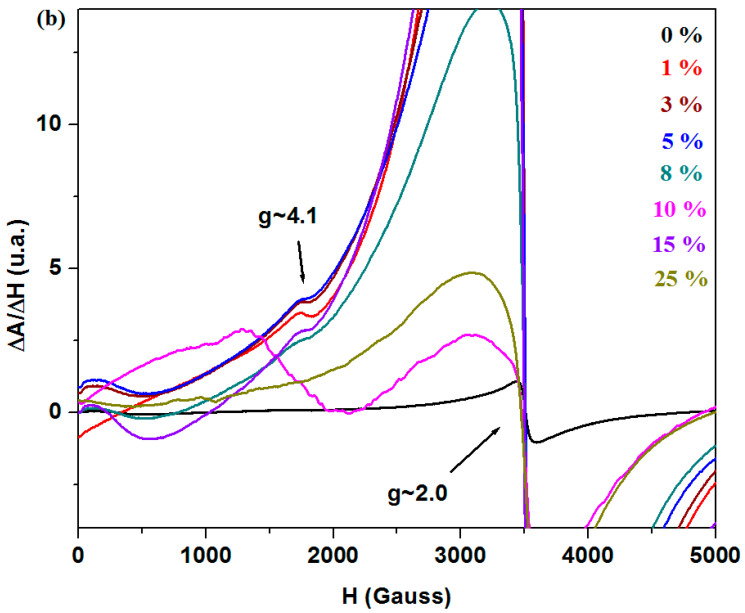
ESR spectra of the recycled vitreous system in the region (**a**) with lower values of the ΔA/ΔH and (**b**) with higher values of ΔA/ΔH.

Electron spin resonance (ESR) spectroscopy is useful for studying paramagnetic systems (with unpaired electrons) and provides detailed information about the local structure and atomic interactions within the sample. The analysis of ionic structure and local interactions within the sample contributes to a better understanding of the electrochemical performance of these materials. This is crucial for the development of more efficient active electrodes in lead-based batteries and other energy-related applications [18]. Iron ions can exist in two valence states, Fe^3+^ and Fe^2+^ ions, but only Fe^3+^ ions (3d^5^, ^6^S_5/2_) generate ESR signals [19]. The ESR spectra obtained for the studied vitreous system with the 3CaO·5Fe_2_O_3_·xFe·(92 − x)Pb composition are presented in Figure 6. The resonance signals observed in the ESR spectra are attributed to the presence of Fe^3+^ ions in the glass-ceramic structure.

The resonance line located at g ≈ 4.1 is attributed to isolated Fe^3+^ ions situated in octahedral and/or tetrahedral coordination sites with rhombic distortion with oxygen atoms. The resonance line at g ≈ 2 is assigned to Fe^3+^ species located in regions with less distorted octahedral/tetrahedral geometries, characterized by lower crystal fields and by the interaction through dipole–dipole interactions, super-exchange mechanisms, or clustering processes [10].

The host matrix exhibits a single resonance line centered at g ≈ 2, which is attributed to clustered Fe^3+^ ions. The ESR spectra of the studied system display two characteristic resonance lines at g ≈ 4.1 and g ≈ 2, indicating that Fe^3+^ ions are present in the glass-ceramic material both as isolated species and as coupled/clustered species.

The analysis of the ESR spectra reveals that the intensity of the resonance line located at g ≈ 4.1, characteristic of isolated Fe^3+^ ions, increases by doping up to 5% Fe, after which it decreases at higher dopant levels, and finally disappears for the sample with 25% Fe. At higher doping levels, the resonance line becomes weak, suggesting a lower number of isolated [FeO_n_] structural units with octahedral/tetrahedral geometry in vitroceramic structure.

The broader signal centered at g ≈ 2.0 is attributed to the formation of clusters of paramagnetic Fe^3+^ ions containing two or more coupled ions [10].

The intensity of the signal located at g ≈ 2 reaches a maximum value at lower Fe doping level of up to 1%, after which it gradually decreases for samples with x ≤ 10% Fe. Beyond this level, at higher dopant concentrations of 15% and 25% Fe, the signal intensity increases again. For the sample with 25% Fe, the signal intensity is weaker compared to the sample with 15% Fe, indicating the conversion of Fe^3+^ into Fe^2+^ ions and an increase in the content of ferrite phase, with the chemical formula Fe_3_O_4_ ≡ Fe^2+^O·Fe^3+^_2_O_3_.

The ESR data indicate that the compositional evolution of the process of isolated/clustered iron ions within the glass-ceramic structure depends on the doping level of the samples.

### 3.5. The Optical Band Gap Energy

The optical band gap energy is the energy difference between the highest energy level in the valence band and the lowest energy level in the conduction band. A larger gap energy increases the energy required to excite electrons from the valence band to the conduction band [20,21]. Materials with an optical band gap energy lower than approximately 1.8 eV are classified as narrow-band-gap semiconductors, while those with a band gap greater than 3.0 eV are considered insulators.

**Figure 7:**
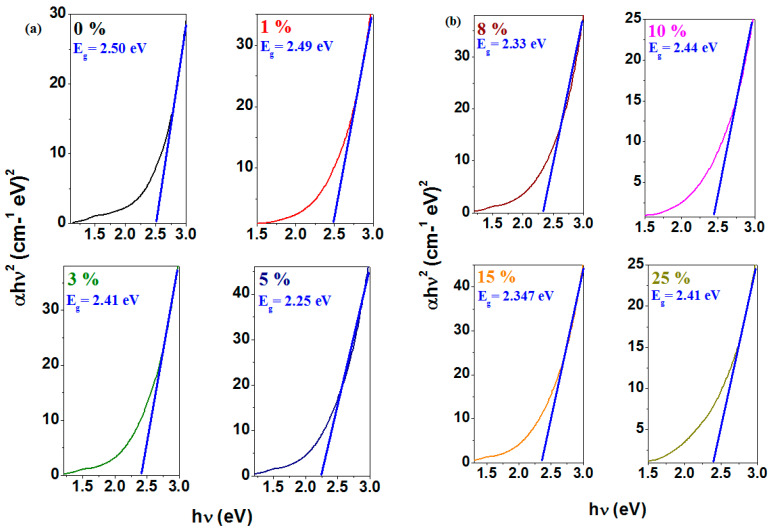

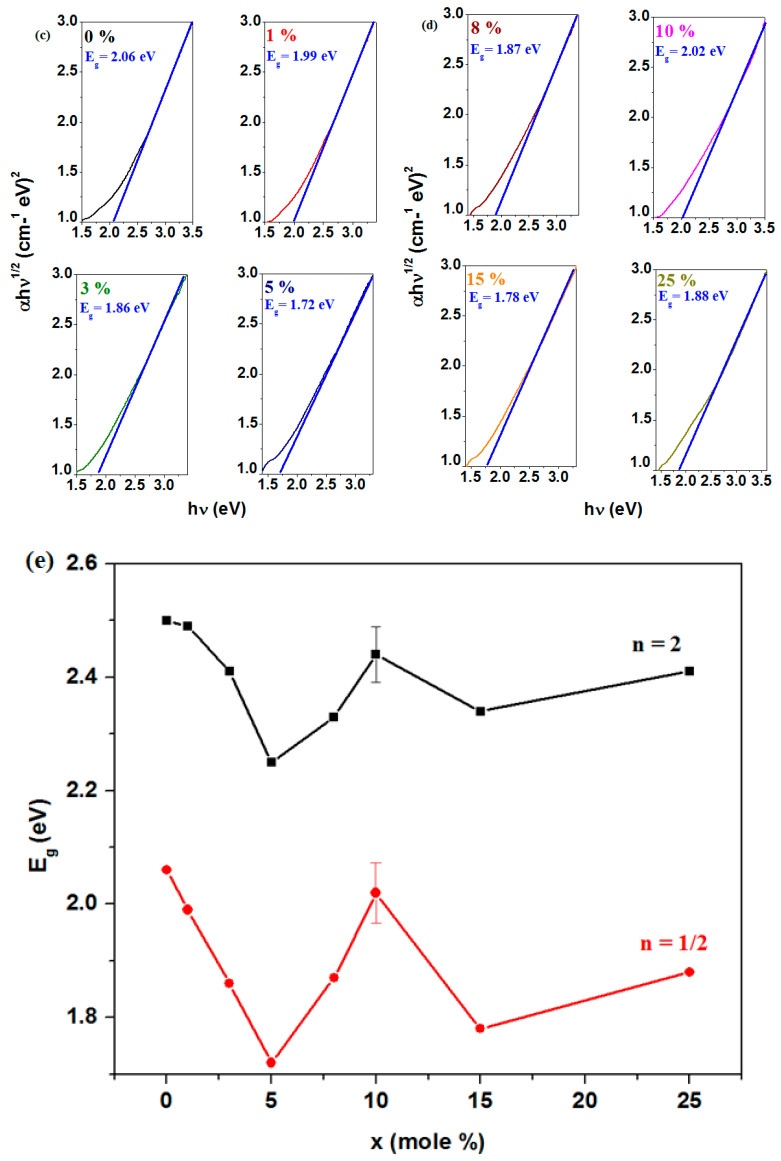
Dependence of (**a**,**b**) (*αhν*)^2^ as a function of *hν* and (**c**,**d**) (*αhν*)^1/2^ as a function of *hν*. (**e**) Extrapolation of the optical band gap energy (*E_g_*) and the compositional evolution of the optical band gap values for direct (*n* = 1/2) and indirect (*n* = 2) transitions in the studied vitreous system.

Two types of transitions, namely direct (*n* = ½) and indirect (*n* = 2) transitions, can occur near the absorption edges of amorphous and crystalline systems. In both cases, electromagnetic waves interact with electrons, allowing transitions across the band gap into the conduction band [22]. The optical band gap energy was calculated using the graphical representation of *(αhν)*^1/2^ or *(αhν)*^2^ as a function of photon energy, *hν*. The band gap energy values, *E_g_*, are determined from the intersection of the horizontal axis with the linear region of these curves. The dependencies of *(αhν)*^1/2^ or *(αhν)*^2^ on photon energy, *hν,* as well as the compositional evolution of the optical band gap energy for direct and indirect transitions of the studied materials, are presented in Figure 7.

The values of the optical band gap energy range from 1.72 to 2.06 eV for direct transitions and from 2.25 to 2.50 eV for indirect transitions. With iron doping in the host matrix, a decrease in the values of band gap energy can be observed, indicating an increase in the density of available electronic states in the sample. This effect is caused by the intercalation of dopants into the glass-ceramic network, which reduces the energy gap between the valence and conduction bands. At lower concentrations, the dopants induce only limited modifications in the electronic structure, which explains the gradual decrease in *E_g_* values.

The *E_g_* values show a slight increase for the sample with x = 10% Fe (2.02 eV for *n* = 1/2 and 2.44 eV for *n* = 2), followed by a decrease to lower values at higher dopant levels. The increase in band gap energy for the sample with 10% Fe suggests a restructuring of the host matrix due to the formation of dopant–rich regions, which create new electronic states. At higher concentrations above 10% Fe, the lower *E_g_* values indicate the accumulation of structural defects. These defects induce states within the band gap that reduce the energy required for electronic transitions.

The undoped host matrix has a value of the highest band gap energy of 2.50 eV, which indicates a well–defined electronic structure and a wide band gap characteristic of more electrically insulating materials. The gap energy decreases as the dopant concentration increases, and the lowest value is evidenced in the sample with x = 5%. All doped samples exhibit an overall reduction in *E_g_* energy, making them more conductive and better suited for electrochemical applications.

This behavior suggests that dopants introduce intermediate states within the energy band of the material that reduce the energies required for electronic transitions.

### 3.6. Cyclic and Linear Sweep Voltammetry Measurements

Cyclic voltammetry is a method in which the applied voltage varies between two limits, a maximum positive potential and a maximum negative potential (−1 V and +1 V), while maintaining a constant scan rate of 10 mV/s. This approach allows for the observation of oxidation and reduction processes and for identification of the electrochemical activity regions of the studied material.

**Figure 8:**
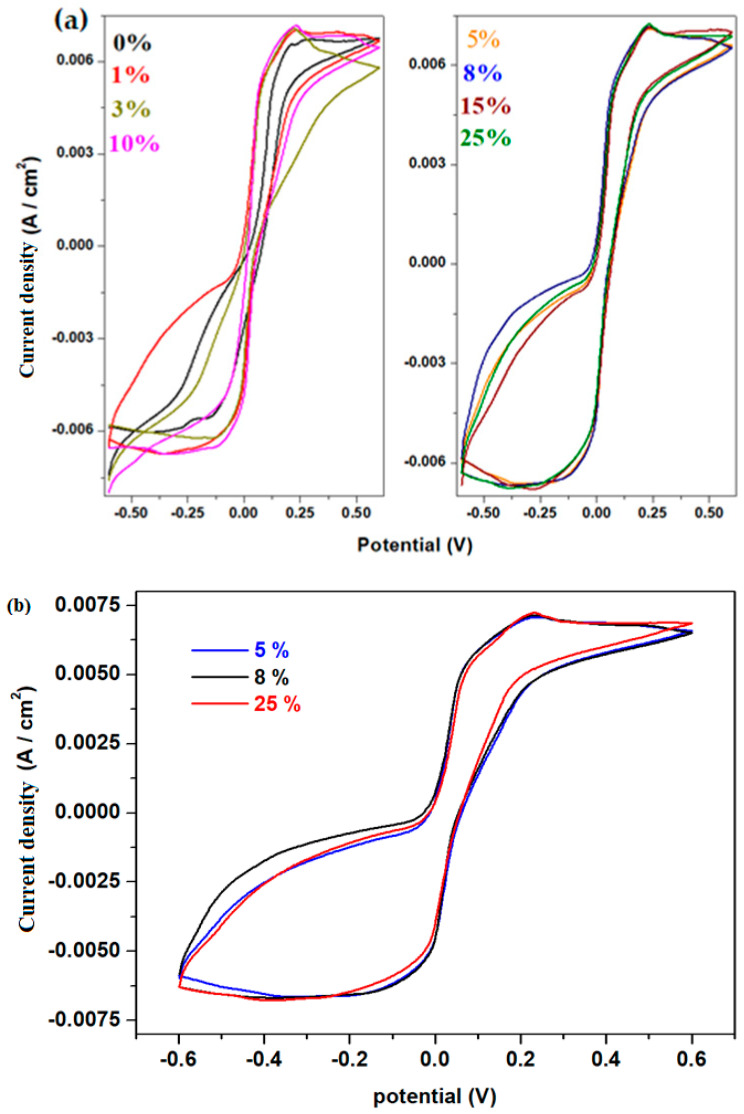
Cyclic voltammograms of the studied materials as working electrode in 5M sulfuric acid solution for (**a**) x = 0–25 % and (**b**) x = 5, 8, 25%.

The cyclic voltammograms recorded with a scan rate of 10 mV/s of the prepared materials are presented in Figure 8.

In the cyclic voltammograms, the oxidation peak is centered around 0.23 V, while the reduction peak appears at −0.13 V. The anodic peak corresponds to two overlapping waves situated at about 0.2 V and +0.28V, associated with the PbO_3_^2−^/PbO_2_^2−^ and PbO_2_/PbO redox systems, respectively. The cathodic peak originates from the Pb^2+^/Pb^0^ redox system [23].

For the samples with 5%, 8%, and 25% Fe, the redox peaks are well defined and become broader.

During scanning, the surface layer of the lead-based anode undergoes a transition from Pb to PbSO_4_ (at −0.356 V), indicating the passivation of the anode surface [24].

**Figure 9:**
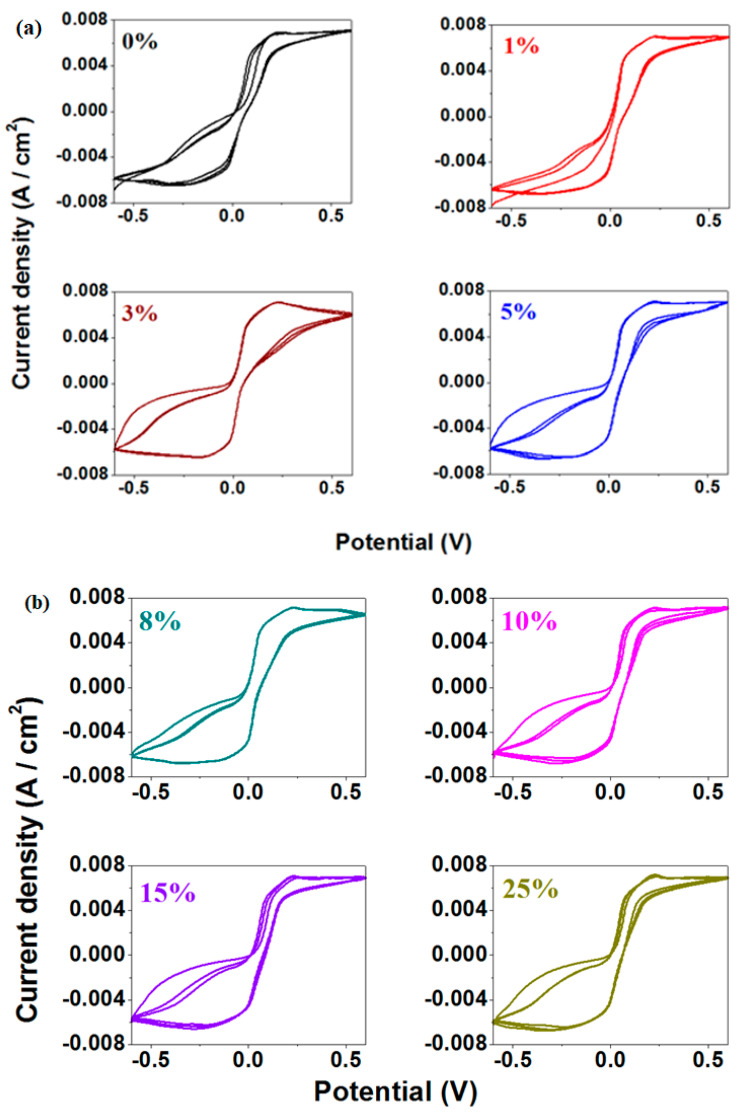

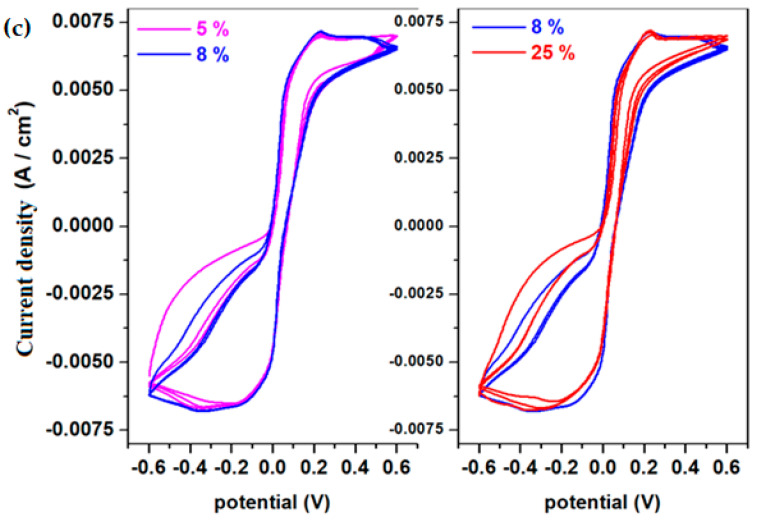
Cyclic voltammograms recorded after three cycles of the studied vitreous system for (**a**) x = 0–5%; (**b**) x = 8–25%, (**c**) x = 5, 8% and x = 8, 25%.

The measurements of cyclic voltammetry recorded after the scanning of three cycles for the studied electrode materials are illustrated in Figure 9. The cyclic voltammograms scanned after three cycles indicate a high degree of irreversibility. For the sample with 8% Fe, the second and third cycles appear to be overlapping quite well.

**Figure 10:**
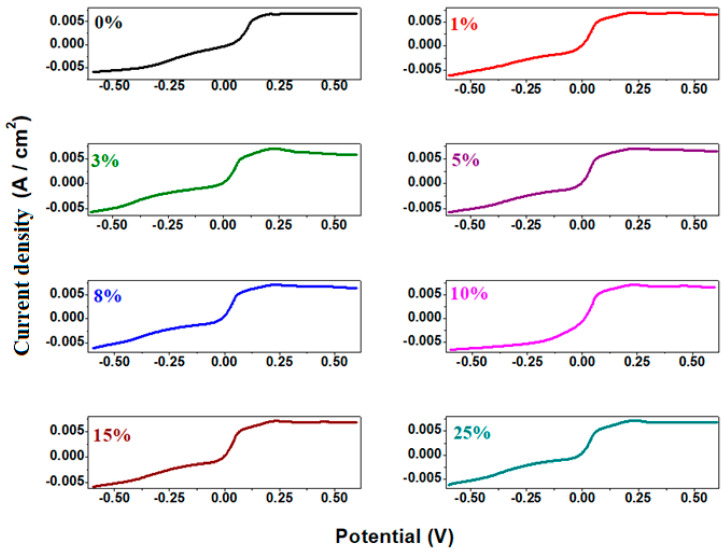
Linear voltammograms of recycled samples.

Linear sweep voltammetry (LSV) is a method that provides additional information about the electrochemical behavior of the material, including the exchange current efficiency and the potential at which electrochemical reactions occur. The measurements of linear sweep voltammetry of the electrode materials in sulfuric acid solution with 5 M concentration are presented in Figure 10.

The electrochemical parameters obtained from cyclic voltammetry and linear sweep voltammetry after scanning one cycle are listed in Table 1. The electrochemical parameters are denoted as follows: E_pA_ represents the anodic potential, I_A_ is the current density of the anodic peak, and E_1/2_ refers to the half-wave potential. It is observed that the intensity of the anodic peak increases with higher dopant concentrations in the host matrix. For the half-wave potential (E_1/2_), lower values are obtained for the samples with x = 8% and 10% Fe, indicating slightly better reversibility compared to their counterparts.

### 3.7. Measurements of Electrochemical Impedance Spectroscopy (EIS)

**Figure 11:**
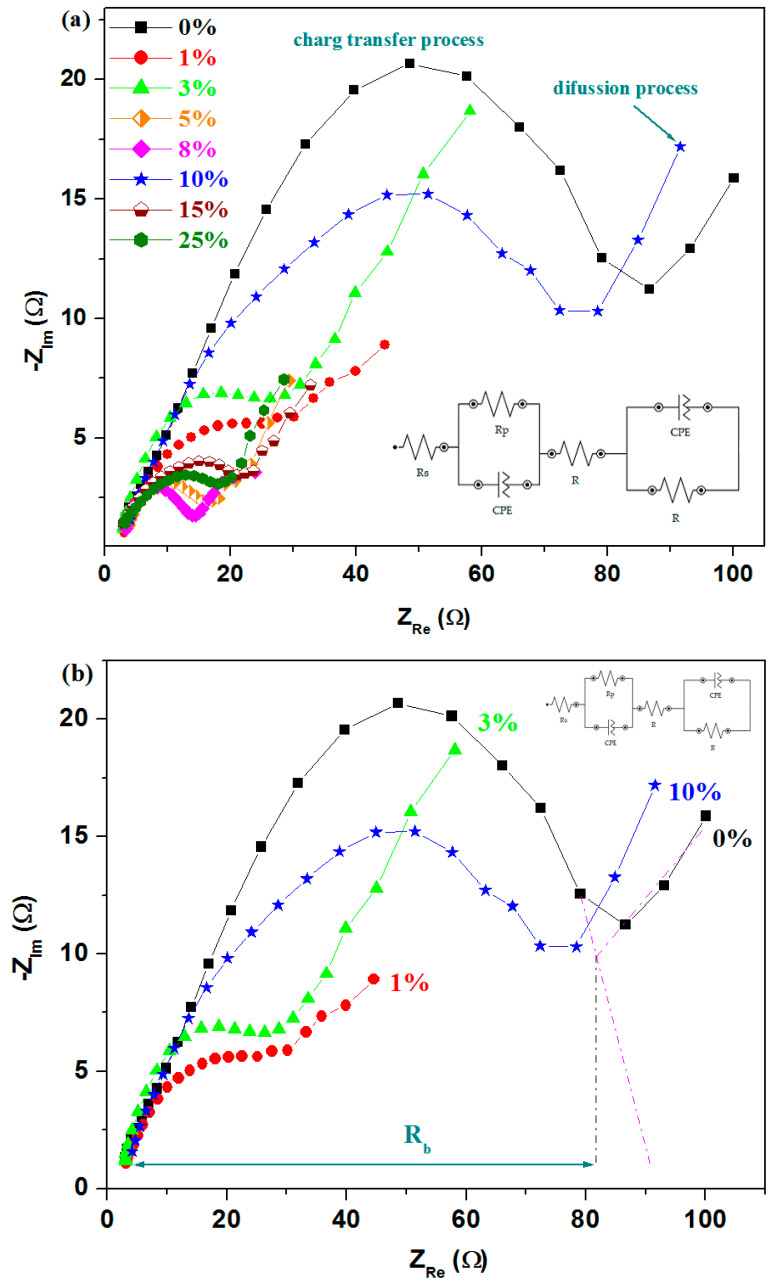

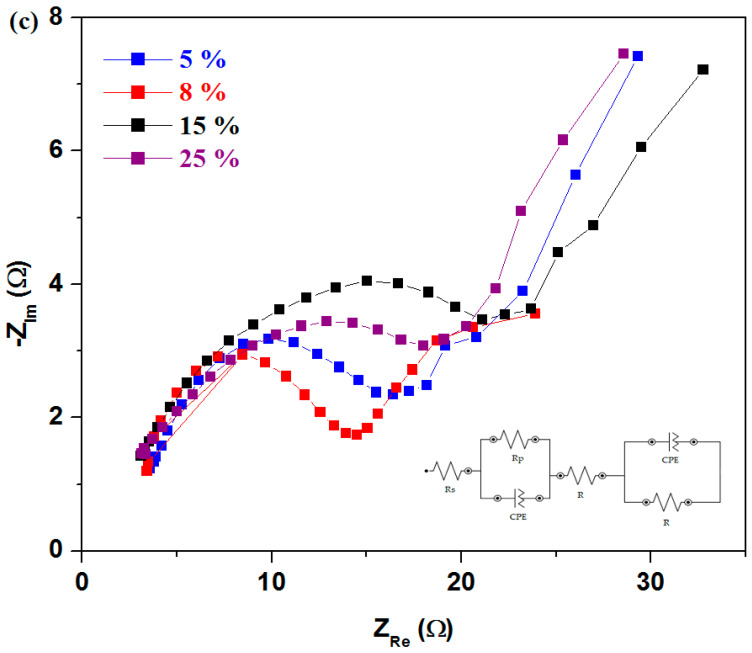
Nyquist plots of complex impedance of the recycled and iron-doped samples for (**a**) x = 0–25 %; (**b**) x = 0, 1, 3, 10; (**c**) 5, 8, 15 and 25%.

The impedance spectra of the studied system and the equivalent circuit used for fitted impedance data are presented in Figure 11.

The equivalent circuit with the [*R*s(*R*_ct_*Q*_dl_)*R*_b_(*Q*_s_*R*_s_)] model type is used in this study for the measurements of EIS to describe complex electrochemical processes. The solution resistance, R_s_, represents the resistance of the electrolyte solution between the reference electrode and the working electrode. The bulk resistance, R_b_, denotes the resistance within the electrode material and reflects its intrinsic electrical properties. The charge transfer resistance, R_ct_, is associated with the charge transfer resistance at the electrode–electrolyte interface during an electrochemical process and provides insight into the kinetics of electrochemical reactions. The double-layer capacitance, Q_dl_, represents the non-ideal capacitive behavior of the electrochemical double layer at the interface [25]. Unlike an ideal capacitor, a constant phase element (CPE) accounts for the frequency dispersion effects observed in real systems. The surface capacitance, Q_s_, represents the capacitive behavior caused by surface phenomena, such as adsorption or surface roughness, which influence the impedance response [14].

The Nyquist plots of complex impedance exhibit a semicircle in the high-frequency region and an inclined slope in the low-frequency region [26]. By doping with a higher iron level, the impedance curves maintain the same shape, but the radius of the semicircle was modified. By increasing the iron content, a decrease in the semicircle radius is evidenced, which indicates the improved conductivity of the studied material. Smaller semicircle radii are observed for the samples with 5%, 8%, 15%, and 25 mol% Fe.

The equivalent series resistance (R_s_) was determined from the tangential intersection of the Nyquist plots on the Z′ axis and represents a measure of the conductivity of the electrode material [27].

**Figure 12:**
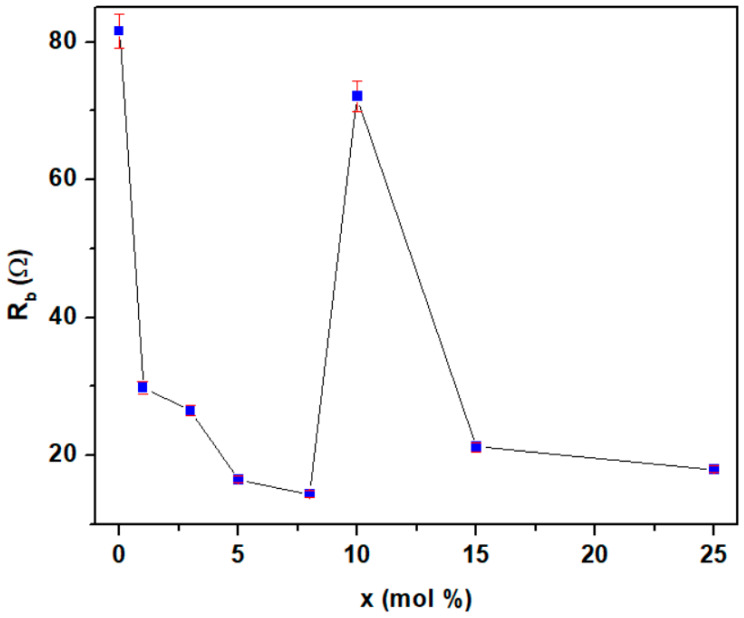
Compositional evolution of material resistance (R_b_) for the studied samples.

The values of material resistances, R_b_, as a function of iron content are presented in Figure 12. The impedance parameters of the material resistance, R_b_, are listed in Table 2. From this graphical representation, the maximum values of the resistances are observed for the samples with x = 0% and 10 mol% Fe. For the sample with 10% Fe, there is a reduction in electrical resistance of approximately 7.23% compared to the reference sample.

Higher values of the material resistance are associated with a lower number of microcracks and with a minimal expansion of the electrode material in an acidic environment. For other samples, a sharp decrease in R_b_ resistance is observed, which is associated with better conductivity in an acidic medium but also with the presence of microcracks in the material. For the undoped sample and the sample with 8% Fe (which has the lowest resistance), the R_b_ resistance decreases from 81.5 Ω to 14.1 Ω. This reduction clearly suggests a higher conductivity for the x = 8% Fe sample, which may be associated with cracks in the material and the expansion effects of the reaction in an acidic environment. All doped materials exhibit better conductivity than the undoped reference sample.

The analysis shows that the samples with 5 and 8% Fe exhibit superior electrochemical properties compared to other compositions, with lower resistance and enhanced electrical conductivity. The lower half-wave potential and lower material resistance suggest that the sample with 8% Fe is the most suitable for application as an electrode in automotive batteries.

On the other hand, from the LSV parameters, it can be seen that the lower values of half-wave potential are found for the samples with 8% (having E_1/2_ = 0.026 V) and 10% Fe (E_1/2_ = 0.021 V), respectively. The value of the current density is somewhat bigger for the sample with 10% compared to its analogue with x = 8%.

**Figure 13:**
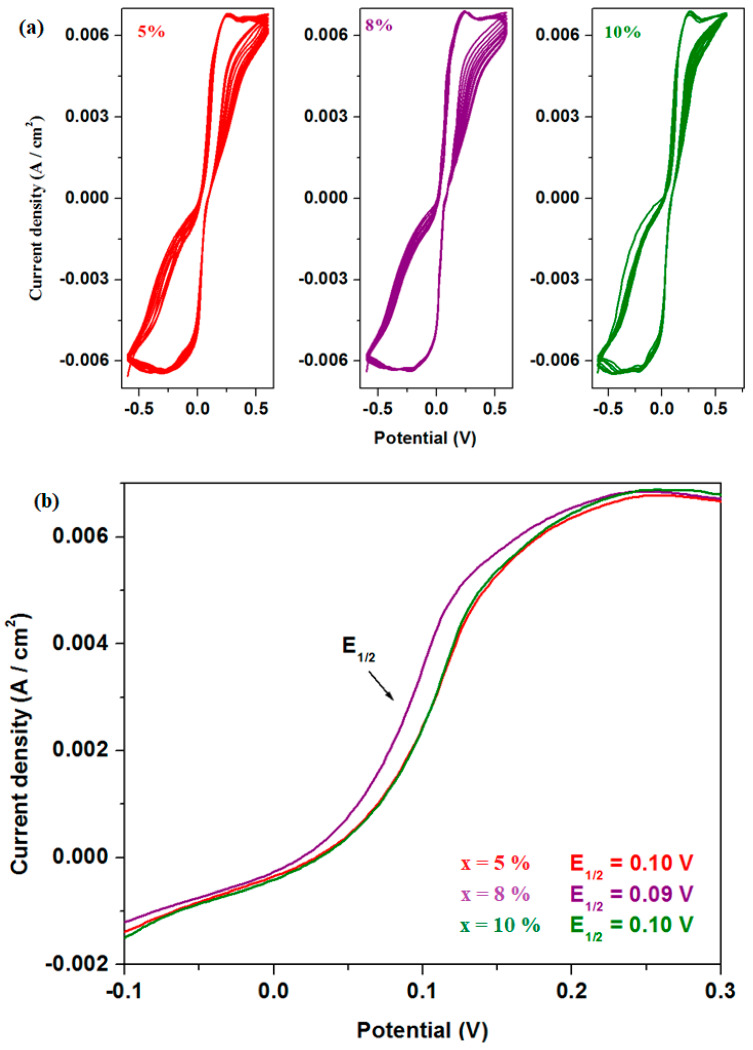
(**a**) Cyclic voltammograms and (**b**) linear voltammograms of recycled samples with x = 5, 8, and 10 mol% Fe recorded after ten cycles.

A comparative study regarding the long-term electrochemical stability of the electrode materials with x = 5, 8, and 10% Fe for lead acid battery applications can be performed through longer cycling tests. The cyclic voltammograms and LSV data after the scanning of ten cycles can be observed in Figure 13. Based on a first interpretation of our results, it could be concluded that the values of E_1/2_ decrease for the sample with 8%, and the anodic intensities are the same for the samples with 8 and 10%. In brief, the longer cycling test also suggests better performance for the sample with 8% Fe.

The use of CaO, Fe_2_O_3_, and Fe compounds in the recycling process of spent batteries represents an efficient and sustainable solution and an important contribution to the optimization of valuable material recovery and the reduction of environmental impact. Due to their beneficial properties, such as availability, non-toxicity, and stability under various oxidation conditions, these compounds play an essential role in improving the safety and efficiency of recycling. Thus, their integration into industrial processes can facilitate the development of more environmentally friendly and responsible methods for the managing of battery waste.

The recycling of anodic plates from lead-acid batteries is an essential process for environmental protection and resource optimization. By recovering and reusing lead and its compounds, pollution is reduced, raw materials are conserved, and the circular economy is supported. Thus, recycling becomes a fundamental component in the responsible management of lead-acid batteries, contributing to a more sustainable future for both industry and the environment.

## 4. Conclusions

The spent anode plate of a discarded car battery was recycled by melt quenching method and was doped with CaO, Fe_2_O_3_, and Fe. Samples obtained with the 3CaO·5Fe_2_O_3_·xFe·(92 − x)Pb composition, where x = 0, 1, 3, 5, 8, 10, 15, and 25 mol%, Fe were investigated by various methods.

The analysis of XRD data indicates vitroceramic structures with predominant phases consisting of lead compounds and ferrite for all samples.

The FTIR spectra reveal the number of sulfate and sulfite units originating from the spent battery plates was decreased by increasing the Fe level. The dopant acts as a network former at lower iron concentrations in the host matrix and as a network modifier at higher levels exceeding 10 mol% Fe. As the dopant content increases, the network modifier breaks PbO–PbSO_4_ bonds from Pb_2_SO_5_ crystalline phase and releases lead oxide, which acts as a network former in the host matrix. Meanwhile, calcium ions replace lead ions in lead sulfate. As a result, a partial desulfatization process of the spent plates occurs.

The UV-Vis spectra indicate electronic transitions attributed to the Pb^2+^, Fe^2+^, and Fe^3+^ ions.

ESR data reveal resonance lines corresponding to the Fe^3+^ ions. At higher dopant levels, the intensity of the resonance lines associated with Fe^3+^ ions decreases, suggesting the conversion of Fe^3+^ to Fe^2+^ ions and the formation of the Fe_3_O_4_ ferrite phase, which contains both Fe^2+^ and Fe^3+^ ions. The values of the optical band gap energy decrease by doping and are below 3 eV, indicating the semiconducting nature of the materials.

The cyclic voltammograms show well-defined peaks, particularly in the anodic region. For the sample with 8 mol% Fe, the lower values of the half-wave potential indicate slightly better reversibility in the voltammogram. The electrochemical impedance spectra also suggest that the sample with 8% Fe exhibits the best conductivity.

In conclusion, the proposed recycling technology for spent automotive battery plates, specifically the melt quenching method, has two major advantages. The first advantage is that it allows for the partial desulfatization of the spent anodic active mass, yielding the partial conversion of lead sulfate phases into lead dioxide and metallic lead. The second advantage is that the newly recycled materials obtained through this method can be used as new cathode electrodes for automotive batteries.

## Figures and Tables

**Table 1:** Electrochemical parameters of the studied samples.

x (mol%)	E_pA_ (V)	E_½_ (V)	I_A_ × 10^−3^ (A/cm^2^)
0	0.21	0.10	6.77
1	0.23	0.029	7.04
3	0.21	0.048	7.13
5	0.22	0.031	7.15
8	0.23	0.026	7.15
10	0.23	0.021	7.25
15	0.22	0.031	7.31
25	0.22	0.029	7.31

**Table 2:** The impedance parameters of the fitted equivalent circuit for the electrode materials.

Sample	R_b_
0%	81.5
1%	29.8
3%	26.5
5%	16.5
8%	14.1
10%	72.1
15%	21.2
25%	18

## Data Availability

The original contributions presented in this study are included in the article. Further inquiries can be directed to the corresponding author.
